# The complete plastid genome of *Aletris megalantha* (Nartheciaceae), an endemic species from Yunnan Province of China

**DOI:** 10.1080/23802359.2020.1866459

**Published:** 2021-01-27

**Authors:** Yong-Chao Wang, Zhi-Rong Zhang, Chao-Nan Fu, Li-Jun Yan

**Affiliations:** a School of Vocational and Technical Education, Yunnan Normal University, Kunming, China; b Germplasm Bank of Wild Species, Kunming Institute of Botany, Chinese Academy of Sciences, Kunming, China; c Key Laboratory for Plant Diversity and Biogeography of East Asia, Kunming Institute of Botany, Chinese Academy of Sciences, Kunming, China

**Keywords:** *Aletris megalantha*, endemic species, Nartheciaceae, phylogeny, plastid genome

## Abstract

*Aletris megalantha* F. T. Wang & Tang is an herbal plant species endemic to Yunnan Province of China. Its complete plastid genome sequence was 154,704 bp in length, with a large single-copy (LSC) region of 83,265 bp, a small single-copy (SSC) region of 18,127 bp, and a pair of inverted repeat regions (IRs) of 26,656 bp. The whole plastid genome encoded 132 genes, including 85 protein-coding genes, 38 tRNA genes, and eight rRNA genes. The overall GC content of *A. megalantha* plastid genome was 37.4%. Maximum likelihood phylogenetic analysis based on 14 taxa indicated that *A. megalantha* is evolutionarily close to *A. spicata*.

*Aletris* is the largest genus in Nartheciaceae, containing about 20 species, which are disjunctively distributed in eastern Asia and eastern North America (Zhao et al. 2012). *Aletris megalantha* F. T. Wang & Tang is an endemic species from Yunnan Province of China (Wu et al. 1997). In the present study, we reported the first complete plastid genome of *A. megalantha* and performed the phylogenetic analysis with other related species within the order of Dioscoreales based on the plastid genome sequences.

Fresh leaves were collected from Yongde county, Yunnan province of China (99°36′54.40″E, 24°3′54.60″N) and the voucher specimen (Voucher number: YDDXSA036) was deposited in the Herbarium of the Kunming Institute of Botany, Chinese Academy of Sciences, Yunnan, China (KUN). Total genomic DNA was extracted from leaf tissue with an improved 4 × CTAB method (Doyle and Doyle 1987). Illumina paired-end (PE) library was constructed, and high-throughput genome sequencing was performed on the Illumina HiSeq X Ten platform. The GetOrganelle v1.7.0 (Jin et al. 2020) and the PGA (Qu et al. 2019) were used to assemble and annotate the chloroplast genome, respectively, with the whole plastid genome sequences of *A. spicata* (NC_033411) as reference. The newly annotated complete plastid genome was submitted to GenBank (accession number MW080684).

The complete plastid genome of *A. megalantha* was 154,704 bp in length, containing a pair of inverted repeats (IRs) of 26,656 bp, a large single-copy (LSC) region of 83,265 bp and a small single-copy (SSC) region of 18,127 bp. The overall GC content of this genome was 37.4% (LSC, 35.3%; SSC, 31.3%; IRs, 42.6%). The whole plastid genome encoded 132 genes, including 85 protein-coding genes, 38 tRNA genes, and eight rRNA genes.

To clarify the phylogenetic position of *A. megalantha*, the complete plastid genomes of 12 species were selected within the order of Dioscoreales. Another two species from the order of Pandanales were chosen as outgroups. The plastomes of the 14 accessions were aligned using MAFFT (Katoh and Standley 2013) plugin in the software PhyloSuite (Zhang et al. 2020). A maximum likelihood analysis was performed using RAxML (Stamatakis 2006) software with GTR + G model and using the rapid bootstrap with 1000 replicates. The genus *Aletris* was supported as a monophyly clade by the phylogenetic analysis, and *A. megalantha* is evolutionarily close to *A. spicata* (Figure 1). The pairwise distance between *A. spicata* and *A. megalantha* was calculated in MEGA X (Kumar et al. 2018) under k-2p model. The pairwise distance (0.0069) indicated that *A. spicata* and *A. megalantha* are genetically different. This report provided a valuable resource for the future studies in *Aletris* and related taxa.

**Figure 1.**
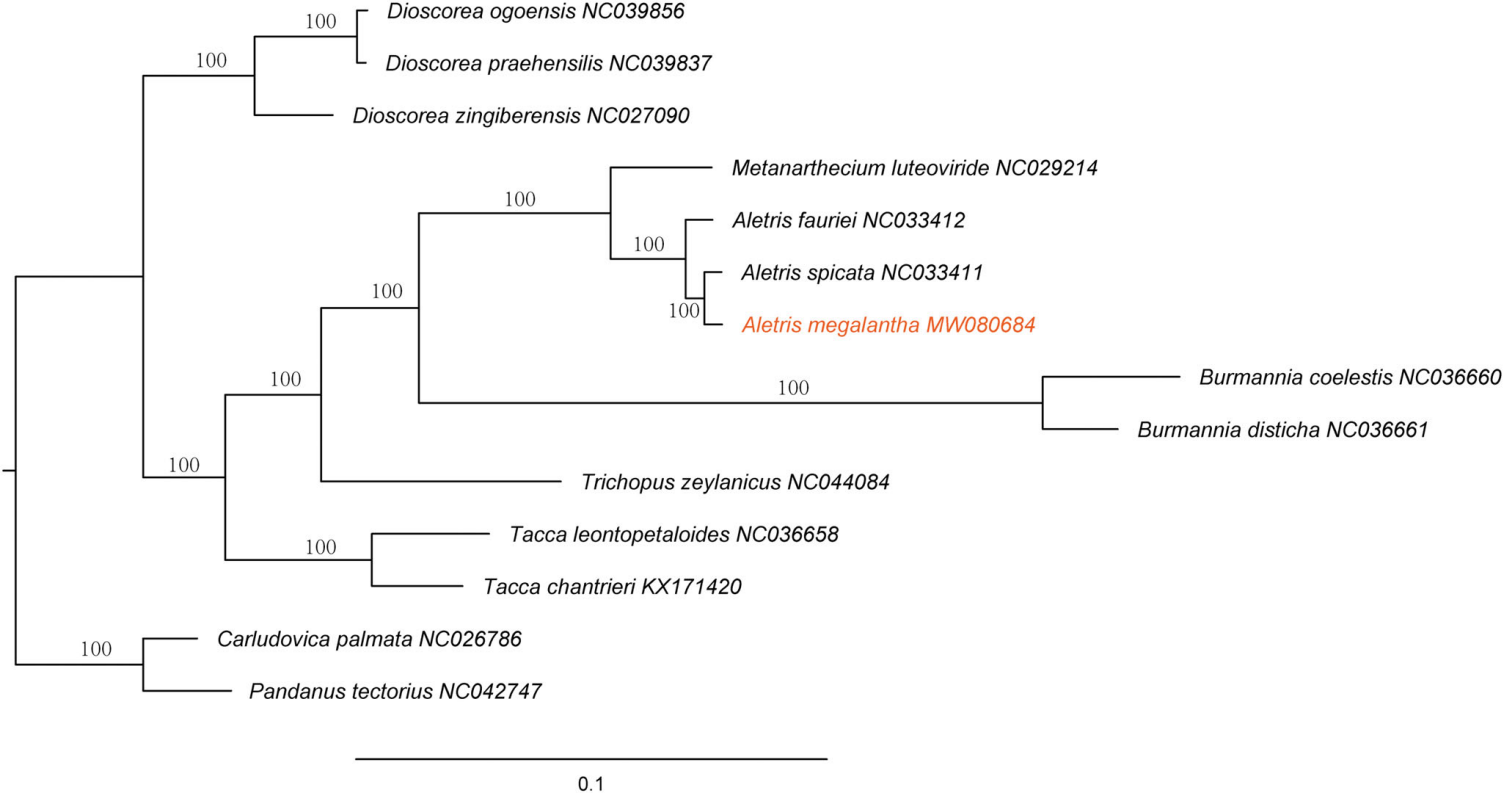
The maximum likelihood (ML) tree based on complete plastid genome sequences from 12 species within the order of Dioscoreales. *Carludovica palmata* (NC026786) and *Pandanus tectorius* (NC042747) were used as outgroups. The bootstrap support values with 1000 replicates are shown at each node.

## Data Availability

The genome sequence data that support the findings of this study are openly available in GenBank of NCBI at (https://www.ncbi.nlm.nih.gov/) under the accession no. MW080684. The associated BioProject, SRA, and Bio-Sample numbers are PRJNA680851, SRR13148561, and SAMN16925232 respectively.
